# Supplementary material for: Effect of insulin treatment on pulsatility ratio and resistance index of the retinal artery in patients with type 2 diabetes
Source: PLoS One. 2021 Jul 20;16(7):e0254980. doi: 10.1371/journal.pone.0254980 (PMC8291657; doi:10.1371/journal.pone.0254980)
Supplement: Attachment — Submitted filename: Response to reviewers.docx [file pone.0254980.s001.docx]

Review Comments to the Author

Please use the space provided to explain your answers to the questions above. You may also include additional comments for the author, including concerns about dual publication, research ethics, or publication ethics. (Please upload your review as an attachment if it exceeds 20,000 characters)

Reviewer #1: The authors are evaluating whether a long-term insulin treatment is associated with abnormalities in retinal circulation in type 2 diabetic patients under the insulin therapy and without the therapy. Quantitative parameters of the measured velocity pulse wave during a cardiac cycle show significant alteration in the retinal arterial circulation in patients with diabetes mellitus type 2. Although the results of the study seem very interesting for clinical applications and retinal blood flow research, the measurement technique does not allow to report directly on the retinal arterial stiffness. Moreover the data analysis and its interpretation flaw.

Major remarks
**Comment 1 :** The title of the paper claims to show effect of insulin treatment on retinal arterial stiffness. However, this supposition of the authors is not directly related to the measurement technique used. The cited references show the method being able to assess velocities in large retinal arteries but it has not ever been validated to measure retinal arterial stiffness itself! The claim of the authors to assess the retinal arterial stiffness on the base of the formulas from the macrocirculation is reasonable but still far-fetched. This reviewer suggests to be honest and to report objectively what was really measured: blood velocities in the retinal artery during the cardiac cycle and related compliance parameters. It is reasonable to mention correctly and carefully in the discussion and probably in the conclusion, that some of these parameters might relate to arterial stiffness/rigidity. However, is too ambitious to postulate this directly in the title. It should be changed and the corresponding ambitious affirmations in the abstract and in the conclusion should be adjusted as well.

**Response 1:** We appreciate the reviewer’s comments and revised the Title, Abstract, and Discussion sections accordingly (Title page 1, line 1, page 2 line 16, page 20 line 8, and line 12).

**Comment 2 :** The discussion section would benefit from more considerations on the etiology and structural changes of the velocity wave form in the retinal arteries and their relation to the stiffness (usually they work with pressure wave form in the PWA). What physiological/ pathophysiological meaning do the waveform changes have? Why the reported parameters and not others were chosen to describe/represent the pulse wave? What do the authors mean with the alteration in the stiffness: a structural remodeling of the arterial wall? A functional constriction of retinal arterioles downstream to the measured arterial segment?

**Response 2 :** We appreciate the reviewer’s comments and have added sentences in the Discussion section accordingly. (page 18, line 6 to 12)

**Comment 3 :** It is advisable to check the correlation of velocity parameters to the vessel diameter. This important interaction is forgotten in Table 5. Even if the vessel diameters were not significantly different in the groups, the insulin group showed smaller diameters and dependent to the flow characteristics the diameter comes in the calculation of the velocity to the power of ~2.

**Response 3 :** We appreciate the reviewer’s comments and have added sentences in the Methods section along with Table 5 accordingly. (page 7, line 3 to 4, line 7, and page 13, Table 5)

**Comment 4 :** The medical ethics issue is questionable: The authors report: “This study complied with the guidelines approved by the ethics Committee of …” Was the study approved or only “complied with”? What institution?

**Response 4 :** We apologize for this confusion and have revised the Discussion section accordingly (page 3, lines 13-15).

Measuring issues:
**Comment 5 :** How accurate the vessel diameter was measured? Was the technique able to measure in µm without correction to the AL that is not done by conventional OCT-devices? The discrepancy to the real value in µm can thus vary in +/- 10%! Additionally the measurement accuracy of 0.1 µm (as reported with the numbers) is incredible for the modern OCT-technology! How was the real precision of the vessel diameter measurement? The issue is very important and can partially explain the unusual controversial result of similar blood flow rates in diabetic and non-diabetic subjects in the study.

**Response 5 :** We appreciate the reviewer’s comments and have revised the discussion section as suggested (page 19, line 23 to page 20 lines 6).

Statistical issues:
**Comment 6 :** The sample sizes of the examined groups are considerably different. The use of t-test is known to be sensible for unequal variances especially for very different sample sizes. Welch’s test should be used instead. The use of ANOVA implies the homoscedasticity as well. As it can be seen from the data, for the key “vascular resistance” parameters in the study the variances are quite different! E.g. the variance of PR is 0.36 in the control group and 4.6 in the insulin group: more than 10 times! (Table 4). Moreover, there are 7 circulatory parameters reported in Tab. 2 with their p-values. To report a solid result on statistical significance one should correct to multiple comparisons here as well (e.g. using Bonferroni correction). The authors are encouraged to apply correct statistical testing, that probably reconsiders their results.

**Response 6 :** We have revised the Tables along with the Methods and Results sections (page 6, line 21, page 8 -9, Table 1, page 9, Table 2, page 10-11,Table 3, Page 12, Table 4).

**Comment 7 :**Line 282: “Typically, an elevated PR or RI indicates decreased vascular compliance and/or increased vascular resistance, distal to the measurement site.[21]”
The affirmation is very general and somehow inaccurate regarding the cited literature, since the pressure-volume relationship, vessel structure and related aspects are different in the microcirculation compared to the macrocirculation. What is meant exactly with “distal to retinal arterioles” that are themselves quite close to the capillary bed?
**Response 7 :** We have revised the Discussion section as suggested (page 15, line 23, and page 16, line 6-10).

Minor remarks:

**Comment 1 :** Line 178: “Pulse wave analysis revealed an increase in the Vmin, but not Vmax, in the diabetic group and a greater PR and RI in diabetic patients.” A confusing affirmation with the view to the results in table 2: Vmin is smaller there in DM group
**Response 1:** We apologize for this confusion. We have revised this sentence accordingly (page 7, line 19).

English of the manuscript should be improved. Some formulations in the manuscript are overall inaccurate and therefore sometimes not understandable, e.g.:

**Comment 2 :** Line 45 and further on: “Retinal vessel rigidity”. Consider „arterial“. The venous rigidity was not measured at all!
**Response 2:** We have revised the manuscript accordingly (page 2, line 6, line 14, and line 18, page 6, line 13, and page 18, line 4).

**Comment 3 :** Line 62: “optimal imaging technology”. What do the authors mean?
**Response 3:** We have revised this phrase in the manuscript (page 2, line 21).

**Comment 4 :** Line 85: “Patients with blood pressure (BP) level…were considered as hypertension.” Or further on: “Patients…. as dyslipidemia.”
Patients cannot be a disease!
**Response 4:** We have revised these sentences (page 3, line 19 to page 4 line 1).

**Comment 5 :** Line 92: “Patients with uncontrolled controlled diabetes.”
What does it mean???
**Response 5:** We deleted controlled in this sentence (page 4, line 5).

**Comment 6 :**Line 95: “a history renal dysfunction or cardiovascular…”
Consider history of!

**Response 6 :** We have revised this sentence accordingly (page 4, line 6).

**Comment 7 :**Line 285: “However, the retinal blood parameter was not significantly different between the insulin and noninsulin groups.”
What retinal blood parameters? Especial parameters of retinal blood?
**Response 7 :** We revised this sentence (page 16, line 1).

**Comment 8 :** Line 96 and others: Articles are frequently missed!
**Response 8 :** We apologize for the missing articles. This has been revised accordingly (page 4, line 9).

**Comment 9 :** Line 258: “Doppler velocimetry also indicated that PR was significantly greater, without significant differences in vessel parameters.”
Quite inaccurate formulation. What are “vessel parameters”? Does the velocity belong to it?
**Response 9 :** Please refer to our revision with regard to this comment in the revised manuscript (page 14, line 20).

**Comment 10 :**Line 286: “Our findings of no significant change in the retinal vessel parameter across these groups…”
In what parameter exactly?

**Response 10 :** We have provided the parameter in the revised sentence (page 16, line 2-3).

Reviewer #2: This article evaluated whether long-term insulin treatment is associated with abnormalities in retinal circulation in type 2 diabetic patients. Overall, the article is well written with interesting findings.
Here are my concerns:
**Comment 1 :** Methods section, subsection subjects - How was IOP measured? Applanation tonometry?

**Response 1:** We apologize for this confusion and have revised the corresponding sentence in the Methods section (page 4, line 10).

**Comment 2 :** Methods section, subsection subjects -The insulin group was defined as those patients receiving insulin therapy for at least 1 year. Although authors mention long term insulin treatment, the duration of insulin treatment was not mentioned. Were they using insulin treatment throughout disease duration?

**Response 2:** We apologize for this confusion and have revised the Methods section (page 5, lines 4-6).

**Comment 3 :** Statistical analysis: The group on insulin treatment very small. Did authors do a formal sample size calculation?

**Response 3:** Preliminary sample size estimation based on initial pilot observations indicated that approximately 20–25 patients should be included in each group in order to ensure 80% power and a significance level of 0.05. Moreover, Miller D.E et al. (*Measurement and Evaluation in Guidance* 1973) recommended that the number of subjects should be 10–15 times the number of variables in multiple regression analysis. Therefore, we believe that the sample size in the insulin (n = 22) and DM group (n = 68) are appropriate for each analysis (Table 6).

**Comment 4 :** Results section: Were confounding factors taken into consideration when evaluating the relationship between PR and insulin use? If yes, please include.

**Response 4 :** We appreciate the reviewer’s comments and have added some sentences in the Discussion section (page 19, line 7-12).

**Comment 5 :** Conclusion section: How will increase in retinal arterial stiffness (owing to long-term insulin treatment) without affecting the diameter, velocity, or blood flow be implicated in the pathogenesis of DR. Please elaborate.
**Response 5:** We appreciate the reviewer’s comments and have added some sentences in the Discussion section (page 18, line 13 to page 19, line 1).

**Comment 6 :** Typo error: Line 94, Delete ‘controlled’ between ‘uncontrolled’ and ‘diabetes’

**Response 6:** We deleted “controlled” in this sentence (page 4, line 5).

**Comment 7 :** Table 1 and 2 not very useful since the information on control group is duplicated in Table 3 and 4.

**Response 7:** Because I believe that it is important that the difference between the whole DM and control groups was shown in Tables 1 and 2, I would like to leave data of the whole DM and control groups in Table 1 and 2, if possible.
